# Influenza surveillance systems using traditional and alternative sources of data: A scoping review

**DOI:** 10.1111/irv.13037

**Published:** 2022-09-08

**Authors:** Aspen Hammond, John J. Kim, Holly Sadler, Katelijn Vandemaele

**Affiliations:** ^1^ Global Influenza Programme, World Health Organization Geneva Switzerland; ^2^ School of Pharmacy University of Waterloo Kitchener Ontario Canada

**Keywords:** correlation, influenza, scoping review, surveillance, syndromic, timeliness

## Abstract

**Objective:**

While the World Health Organization's recommendation of syndromic sentinel surveillance for influenza is an efficient method to collect high‐quality data, limitations exist. Aligned with the Research Recommendation 1.1.2 of the WHO Public Health Research Agenda for Influenza—to identify reliable complementary influenza surveillance systems which provide real‐time estimates of influenza activity—we performed a scoping review to map the extent and nature of published literature on the use of non‐traditional sources of syndromic surveillance data for influenza.

**Methods:**

We searched three electronic databases (PubMed, Web of Science, and Scopus) for articles in English, French, and Spanish, published between January 1 2007 and January 28 2022. Studies were included if they directly compared at least one non‐traditional with a traditional influenza surveillance system in terms of correlation in activity or timeliness.

**Findings:**

We retrieved 823 articles of which 57 were included for analysis. Fifteen articles considered electronic health records (EHR), 11 participatory surveillance, 10 online searches and webpage traffic, seven Twitter, five absenteeism, four telephone health lines, three medication sales, two media reporting, and five looked at other miscellaneous sources of data. Several articles considered more than one non‐traditional surveillance method.

**Conclusion:**

We identified eight categories and a miscellaneous group of non‐traditional influenza surveillance systems with varying levels of evidence on timeliness and correlation to traditional surveillance systems. Analyses of EHR and participatory surveillance systems appeared to have the most agreement on timeliness and correlation to traditional systems. Studies suggested non‐traditional surveillance systems as complements rather than replacements to traditional systems.

## 1 INTRODUCTION

Globally, influenza is estimated to cause 290,000 to 650,000 respiratory deaths every year.^1^, ^2^ In 2019, the World Health Organization (WHO) declared an influenza pandemic as one of the top 10 threats to global health.^3^ In order to prepare for and mitigate the consequences of influenza epidemics and pandemics, WHO has conducted, since 1952, influenza virological surveillance through its Global Influenza Surveillance and Response System (GISRS), formerly known as the Global Influenza Surveillance Network (GISN).^2^ During the 2009 influenza pandemic, it became apparent that a standardized approach to epidemiological surveillance for influenza was needed, and thus, the first Global Epidemiological Surveillance Standards for Influenza were developed.^4^ The overarching goal of influenza surveillance is to minimize the impact of influenza through providing accurate and timely information to public health authorities such that they can better prepare for and respond to influenza outbreaks through vaccination and other public health interventions.^4^ Both syndromic and virological data are needed, as while syndromic surveillance can capture trends and offer epidemiological context, virological data are important for understanding the specific contributions of influenza viruses to respiratory disease and to inform vaccine strain selection.

The 2014 WHO Global Epidemiological Surveillance Standards for Influenza recommend a “sentinel surveillance” system which involves routine collection of epidemiological and virological data from a limited number of surveillance sites.^4^ While this is perhaps the most efficient method to collect high‐quality data from a resource perspective, several limitations are undoubtedly present. First, these systems only capture individuals infected with influenza who seek healthcare, reflecting only a portion of the actual disease burden. Additionally, there is a delay between the onset of symptom and healthcare seeking, with a further lag of 1 to 2 weeks between data collection and reporting. Third, an effective sentinel surveillance system relies on several factors such as good laboratory facilities and well‐qualified staff which are features that are not always feasible.^5^ Fourth, one of the criteria in selecting a sentinel site is that the facility should serve a relatively large population that has easy access to it which inevitably introduces a degree of sampling bias.^5^

To address the limitations of the currently recommended surveillance systems for influenza illness, there has been a global effort in developing influenza surveillance systems which use alternative sources of data such as internet search query data or absenteeism data. But without a gold standard measurement of influenza incidence to compare against, it is difficult to understand which systems are working well and the concurrence between various systems.^6^ Additionally, the literature is scattered and the landscape of the current knowledge is difficult to assess. In this study, we aim to map published literature on the use of non‐traditional sources of influenza surveillance data and examine the extent, range, and nature of published literature on the correlation of surveillance systems using traditional and alternative sources of data. The ultimate objective, aligning with the Research Recommendation 1.1.2 of the WHO Public Health Research Agenda for Influenza, is to identify the reliability of complementary influenza surveillance systems to provide real‐time estimates of influenza activity.^7^

## 2 METHODS

A detailed study protocol was developed a priori in accordance with the Joanna Briggs Institute's Reviewer's Manual.^8^ JK, AH, and KV reviewed and approved the protocol prior to the start of this project. The protocol was not published nor registered in any online database.

### 2.1 Inclusion criteria

#### 2.1.1 Types of participants

This scoping review considered anonymized, aggregated results from surveillance for influenza. Studies involving animals were excluded from the review.

#### 2.1.2 Concept

The core concepts examined in this scoping review were influenza surveillance systems using sentinel influenza‐like illness (ILI) and severe acute respiratory infection (SARI) data, as recommended in the WHO Standards (termed traditional) and complementary systems using alternative data such as internet search query data and electronic health records (EHR) (termed non‐traditional). The focus of the review is to identify published papers that compare traditional and non‐traditional surveillance systems on timeliness and correlative strength between traditional and non‐traditional surveillance systems.

Studies that considered percent positivity for influenza, influenza cases, or ILI or SARI as syndromic indicators of influenza illness and considered either of the primary outcomes listed below were included in the review:

- *Correlation/concurrence*: How well does the system correlate with the traditional surveillance method regarding the timing of the start and the peak of an epidemic and the peak intensity?
- *Timeliness*: Is the system able to provide real‐time data? Is the system able to detect the start of or peak activity before traditional surveillance?

#### 2.1.3 Geographical context

This scoping review did not impose any restrictions on the geographic setting of the studies. It considered studies that had been conducted in any healthcare and non‐healthcare settings including, but not limited to, pharmacies, hospitals, long‐term care facilities, and educational institutions.

#### 2.1.4 Types of studies

This scoping review considered published studies of all types, except reviews. However, studies included in systematic reviews were considered for eligibility in this scoping review. Modeling studies looking only at nowcasting or forecasting were also excluded from the review.

### 2.2 Exclusion criteria

Studies meeting any of the following criteria were excluded:

- Not published in English, French, or Spanish
- Published in 2006 or earlier
- Studies not published in a peer‐reviewed journal
- Studies reporting on animal surveillance
- No or inadequate details on characteristics of influenza surveillance systems included (biases, case definitions, etc.)
- Studies that considered only Google Flu Trends as the non‐traditional system (as it is no longer in operation)
- Nowcasting or forecasting studies
- Reviews

### 2.3 Search strategy

The search strategy looked for studies published in English, French, or Spanish. The search was conducted on PubMed, Web of Science, and Scopus on July 2, 2019 and then updated on January 28, 2022 to ensure recent publications were included. Two additional studies were manually identified from the reference lists of retrieved articles. A full description of the search strategy is shown in Table S1 in the supporting information.

Four key themes of the review question were identified. Search terms were combined using “OR” within each theme and “AND” between themes.

1. Surveillance methods
2. Correlation
3. Timeliness
4. Influenza

### 2.4 Study selection

Multiple iterations of the search strategies were employed throughout the review as we explored and focused more narrowly on the review question. Retrieved studies were imported into Covidence,* a systematic review management tool, to automatically remove duplicate studies and to streamline the study selection process.

Three reviewers (JK, AH, and HS) independently screened titles and abstracts for eligibility and appropriateness of the studies. Full texts of eligible studies were then screened for inclusion for data extraction, and duplicate studies not identified by Covidence were removed. For all studies where discrepancies arose in study selection, two reviewers discussed the issue to reach a consensus. If the issue was not resolved following the discussion, a further reviewer (KV) could be consulted to reach a decision. For studies not presented in English, Google document translator was utilized.

### 2.5 Data extraction

Two reviewers (JK and HS) independently reviewed and extracted data from eligible studies using the data extraction tool presented in the appendix. The extraction tool was developed based on the template provided in the Joanna Briggs Institute Reviewer's Manual then further modified to align with our review question.^8^ Results of the initial data extraction were reviewed by a second reviewer (AH) for accuracy and comprehensiveness. Any discrepancies in the data extraction process could be discussed by the reviewers, and a further reviewer (KV) could be consulted if the issue is unresolved. Following data extraction, data were synthesized and illustrated using both narrative and graphical methods.

## 3 RESULTS

### 3.1 Selection of literature

We identified 823 articles from PubMed, Web of Science, and Scopus published between January 1, 2007 and January 28, 2022, of which 138 duplicates were removed. A total of 785 articles were identified as candidates for review of titles and abstracts. A total of 627 articles were removed due to irrelevancy, and 158 full‐text articles were assessed for eligibility. After the full‐text review, 101 articles were excluded. Finally, 57 articles were included for data extraction and analyses. However, many articles considered multiple surveillance systems, were conducted in multiple geographical locations, or performed over multiple seasons so that in our analyses the number of articles by geographical location, year, or system type sums to more than 57.

### 3.2 Geographical distribution

The 57 articles encompassed 22 geographical locations. Over one third of articles were conducted partially or entirely in the United States of America (USA) (*n =* 21), and approximately two thirds were conducted in the four most prevalent geographical locations: the USA, China (*n =* 8), Canada (*n =* 5), and Spain (*n =* 5). A full list of the distribution of the literature by geographical location is shown in Table 1.

**TABLE 1:** Distribution of included literature by geographical location

Geographical location	No. of articles (*n =* 72)
Australia	2
Austria	1
Belgium	4
Canada	5
China	8
Denmark	1
France	3
Germany	1
Ireland	1
Italy	2
Korea, Republic of	3
Malta	1
Mexico	1
Netherlands	3
Portugal	1
Singapore	1
Slovenia	1
Spain	5
Sweden	3
Taiwan, China	2
United Kingdom	2
United States of America	21

*Note*: As several studies were conducted in multiple geographical locations, the total number of articles here sums to 72.

### 3.3 Temporal distribution

**FIGURE 1:**
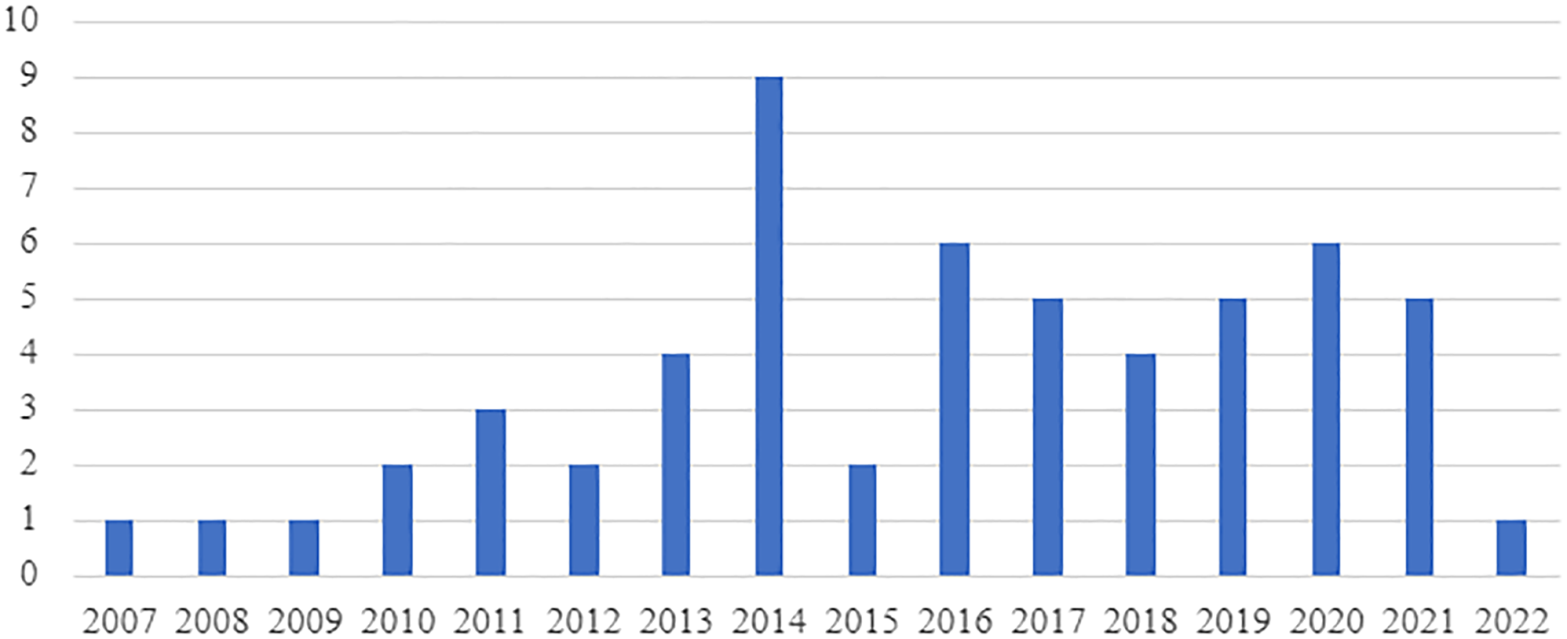
Number of articles by year of publication

**FIGURE 2:**
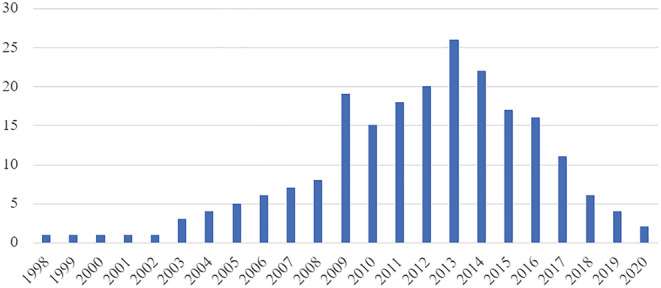
Number of articles by years in period of study

An upward trend was observed in the number of publications per year from 2007 to a peak in 2014, after which (with the exception of 2015 when there were few publications) the number of publications remained relatively stable (Figure 1). There was an increase in the number of articles studying each year until 2013, after which the frequency started to decrease (Figure 2). Year 2013 was the most studied year and notably includes studies performed during the 2013–2014 season which was the first A(H1N1)pdm09‐predominant season in the United States after the 2009 pandemic.^9^ Year 2009 was also well‐studied compared with neighboring years.

### 3.4 Surveillance systems by data source

**FIGURE 3:**
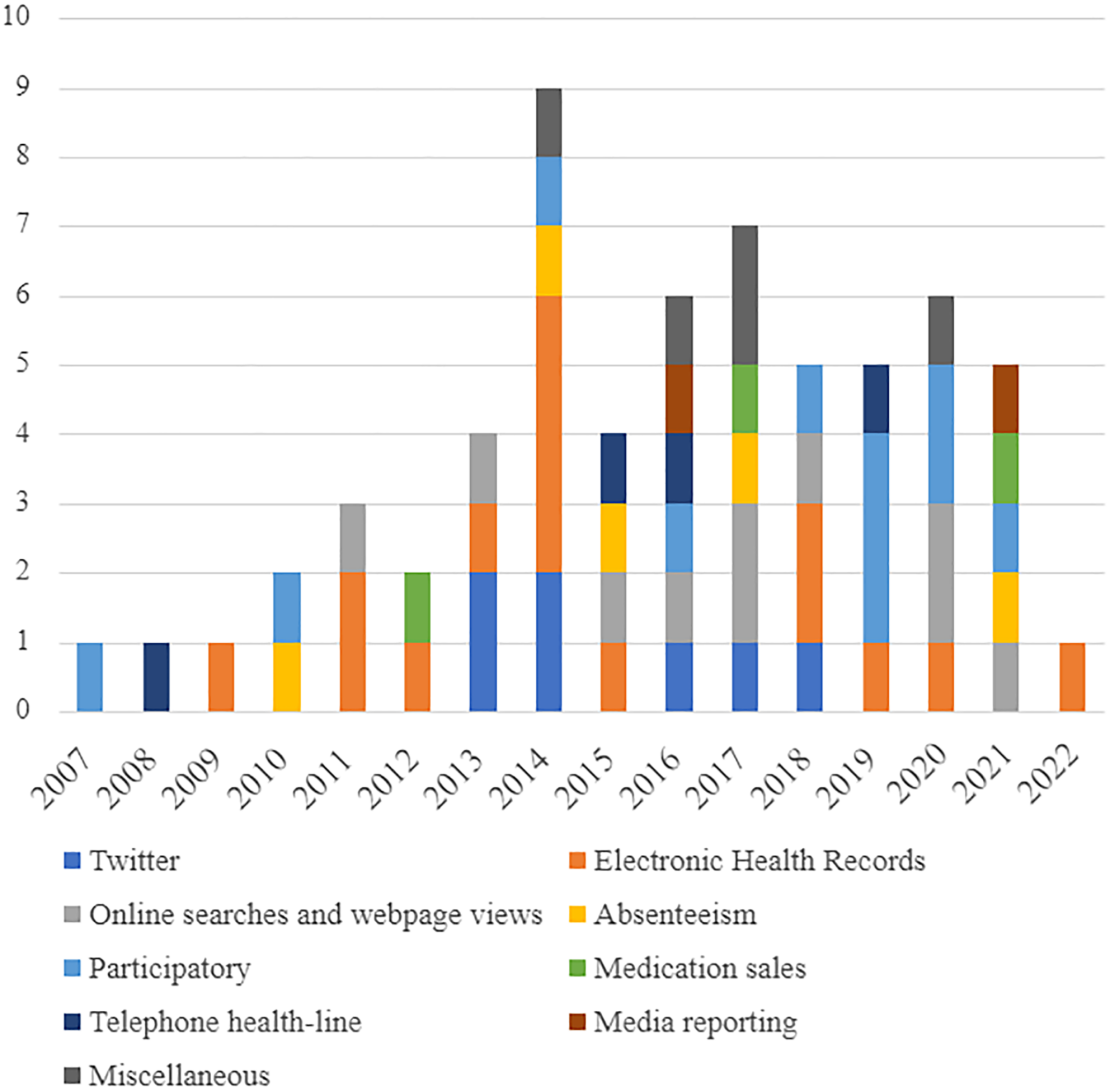
Alternative surveillance systems by data source and publication year. As several articles address multiple data source categories, the number of surveillance systems is different to the total number of articles and sums to 62.

The non‐traditional surveillance systems were placed into nine categories: EHR‐based systems were the most commonly studied (*n =* 15, 42.4%), followed by participatory survey (*n =* 11; 31.1%), online searches and webpage traffic (*n =* 10; 28.3%), Twitter (*n =* 7; 19.8%), absenteeism (*n =* 5; 14.1%), miscellaneous (*n =* 5; 14.1%), telephone health line (*n =* 4; 11.3%), medication sales (*n =* 3; 8.5%), and media reporting (*n =* 2, 5.7%). The surveillance systems are plotted by data source category and publication year in Figure 3, and details regarding the surveillance systems considered in each study are summarized in the supporting information (Table S2).

Of the 57 articles included in the review, 36 and 46 studies considered the timeliness and correlative value of the non‐traditional surveillance systems compared with the traditional system, respectively.

#### 3.4.1 EHR‐based surveillance system

EHR‐based surveillance system studies were most frequently found in our search. Among the 15 articles, nearly all supported EHR‐based systems as a potential complement to the traditional surveillance system.^10^, ^11^, ^12^, ^13^, ^14^, ^15^, ^16^, ^17^, ^18^, ^19^, ^20^, ^21^, ^22^, ^23^, ^24^ There appears to be a general consensus in the literature suggesting that EHR‐based surveillance systems may be both timely and correlate strongly with sentinel surveillance data such that they may be implemented as a complementary system.

EHR‐based surveillance systems were typically found to track with or lead traditional ILI and virological surveillance by up to around 1 week. In contrast, De Oliveira Bernardo et al. found that the Australian sentinel general practitioner (GP) network ASPREN detected an earlier increase in ILI rate than EHRs, though this may have been due to presentations for non‐influenza respiratory viruses at sentinel sites.^16^

#### 3.4.2 Participatory survey‐based surveillance system

Our search found 11 studies looking at participatory survey‐based surveillance system.^12^, ^25^, ^26^, ^27^, ^28^, ^29^, ^30^, ^31^, ^32^, ^33^, ^34^ Although different variations of participatory surveys were considered, we observed that most studies identified participatory survey‐based systems to potentially be a reliable complement to the current sentinel surveillance system. However, Lwin et al. found a correlation which was weaker than in most other studies between ILI symptoms reported by a targeted group of healthcare workers through a mobile app and national ILI incidence in primary care in Singapore.^28^

Two articles addressed timeliness. Rehn et al. found that ILI reports from an internet‐based monitoring system in Sweden correlated most strongly with laboratory data when no time lag was used for both influenza seasons under study (2011–2012 and 2012–2013)^31^. They also correlated optimally with no time lag with ILI‐reporting data from a well‐established and rigorously evaluated population‐based system (which unlike the internet‐based monitoring system uses randomly selected participants). For the 2012–2013 influenza season, they also investigated the correlation between the internet‐based monitoring system and GP sentinel data on ILI cases and found the strongest correlation with GP sentinel data shifted 1 week back in time. Van Noort et al. collected data through voluntary online reports (Gripenet) and found that ILI activity rose, peaked, and declined simultaneously with data from sentinel networks collected by the European Influenza Surveillance Scheme (EISS) for the 2006–2007 influenza season for Belgium, the Netherlands, and Portugal.^33^

#### 3.4.3 Online searches and internet traffic

Our search identified 10 articles addressing internet search queries or webpage views.^35^, ^36^, ^37^, ^38^, ^39^, ^40^, ^41^, ^42^, ^43^, ^44^ Four articles studied Baidu, a popular internet search engine in China.^38^, ^39^, ^41^, ^44^ Three of these provided data on both correlative value and timeliness. Yuan et al. found a very strong correlation between influenza‐related search terms and nationwide influenza case count data and observed that this was maximized when no lag was incorporated.^44^ Dong et al. identified significant correlations between Baidu searches for influenza‐related terms and sentinel hospital virological and ILI data in the city of Tianjin.^38^ Optimal correlations with virological data were obtained for different terms by using no lag or by shifting the data by 1 week in either direction. Liang et al. looked at the correlation between 46 influenza‐related search terms in Baidu and influenza cases in Liaoning province.^41^ They described strong correlations for 17 of these terms, and some of these correlations were highest when a lag of 1, 2, or 3 months was applied.

Two articles studied searches made through a Swedish medical website (Vårdguiden). Hulth et al. showed that searches peaked 1 week before ILI consultations at sentinel GPs.^40^ A second study by Ma et al. identified that influenza‐related data from this website peaked 1 or 2 weeks in advance of sentinel ILI and laboratory surveillance systems for three out of four influenza seasons studied and 1 or 2 weeks behind in the other.^42^ Strong positive correlations were observed between the search data and both traditional surveillance systems for all four seasons.

Two articles looked at the correlation between Google Trends data and traditional surveillance systems. Chang et al. found that influenza‐related searches correlated strongly with reported ILI emergencies and influenza incidence, with very similar correlations seen when a 1‐week lag was modeled.^36^ An Australian study compared Google Trends data with the notifiable disease data for influenza cases and found moderate correlations for some states and weak correlations in others.^43^

We found two further studies which used webpage view data rather than search terms to look at internet behavior. De Toni et al. found that page views for influenza‐related Wikipedia pages in four European countries correlated well with ILI incidence data for each country.^39^ Caldwell et al. evaluated internet traffic to influenza‐related pages on the US Centers for Disease Control (CDC) website against the percentage of visits for ILI across three influenza seasons.^37^ They found strong correlations for some seasons, particularly at national and regional levels but no correlation in many other cases, particularly when looking at the state level. Depending on which pages were considered, the geographical unit and the season under study, optimal correlations were achieved either with no lag or using ILI data 1 week in advance of online search data. These mixed results indicate that these data must be better understood before they can be successfully used to enhance influenza surveillance.

#### 3.4.4 Twitter‐based surveillance system

Our search identified seven studies comparing traditional sentinel surveillance to Twitter‐based surveillance systems.^45^, ^46^, ^47^, ^48^, ^49^, ^50^, ^51^ The evidence supporting Twitter‐based surveillance systems were conflicting. Four papers from the USA and one from the Republic of Korea found strong correlations between Twitter and influenza activity.^45^, ^46^, ^47^, ^49^, ^51^ Of these, only Aslam et al. addressed the timeliness of the system, showing that sentinel and emergency department ILI rates and Twitter activity increased and decreased at approximately the same time for all but one US city under study.^46^

Two further studies found weaker correlations which also varied by location.^48^, ^50^ Nagel noted that in some US cities, Tweet rate peaked before ILI activity, while in others ILI activity peaked first or at a similar time to Twitter activity. Volkova found that Twitter activity in US military communities led or lagged behind ILI activity by up to 4 weeks and that this varied by Tweet theme.

#### 3.4.5 Absenteeism‐based surveillance system

Five absenteeism‐based surveillance system studies were identified in our search.^38^, ^42^, ^52^, ^53^, ^54^ We did not observe concurrence in results between the studies. Four of the articles used absenteeism data from educational settings. Fan et al. found that a school absenteeism system (SAS) identified ILI outbreaks 2 to 4 days in advance in rural China.^54^ They reported that SAS may be an important and feasible system in proactively managing infectious disease outbreaks. A second study conducted in China suggested that school absenteeism data were able to identify influenza activity 1 week earlier than laboratory surveillance and with greater geographic specificity.^38^ However, the correlation with traditional laboratory‐confirmed surveillance system was only moderate. Meanwhile, Ma et al. found the performance of SAS in Sweden to be inconsistent and inaccurate.^42^They concluded that the performance of SAS to be insufficient to be considered as an early notification system. Barrientos found that absenteeism for ILI in kindergartens, primary, and secondary public schools in Spain peaked 2 weeks in advance of peaks in influenza detections through notifiable disease reporting and the sentinel network but did not quantify the strength of the correlation between the surveillance systems.^52^

Duchemin et al. evaluated the correlation between workplace sick leave data and ILI rates in France and found that peaks in sick leave occurred several weeks before peaks in ILI incidence and triggered elevated activity alerts earlier than the sentinel surveillance system.^53^

#### 3.4.6 Telephone health line‐based surveillance system

Our search found four studies considering telephone health lines as a syndromic surveillance system.^42^, ^55^, ^56^, ^57^ Three studies considered systems with medical hotline or telephone health helpline, while one study looked at calls made to an emergency medical service (EMS) call center.

There did not appear to be a clear consensus between the four studies regarding the timeliness and correlative strength of a telephone health line‐based surveillance system. Arcos Gonzalez et al. found that EMS call centers were able to detect the peak of the seasonal influenza 1 week in advance, but the correlation with the traditional surveillance system was only weakly statistically significant.^55^ A study from Sweden concluded that while the medical hotline system correlated strongly with the traditional surveillance system, it did not perform with enough consistency to be confidently considered as an early notification system.^42^ Two studies from Canada provided positive outcomes with their system's ability to be used as a real‐time syndromic surveillance system as a complementary tool.^56^, ^57^

#### 3.4.7 Medication sales‐based surveillance system

We identified three studies looking at over‐the‐counter (OTC) and prescription medication sales for colds and acute respiratory tract infections as a method of surveillance.^38^, ^58^, ^59^ In two of these, the correlation between medication sales and routine surveillance appears to only be moderate.^38^, ^59^ Of these, only Dong et al. addressed timeliness, identifying that the correlation was highest when compared with ILI data 1 week in advance.^38^ Conversely, Choi et al. found a strong correlation between the proportion of ILI‐related drug claims and the ILI rate from clinical sentinel surveillance reported by the Korean Center for Disease Control (KCDC) for all influenza seasons between 2014 and 2018.^58^ No lag was found between the claims data and the sentinel data from KCDC.

#### 3.4.8 Media reporting

Two articles addressed the use of media reports.^60^, ^61^ Yan et al. found that there was a statistically significant moderate correlation between influenza‐related news items and the number of new hospital notifications in China during the 2009 influenza pandemic and that this was highest when a 4‐day lag was applied.^61^ Li et al. considered whether official and unofficial online news articles could be used to improve forecasting models for ILI during the 2019–2020 influenza season.^60^ While their principal investigations fall outside the scope of this review, they identified that the peak in influenza‐related online news articles and microblogs preceded the peak in ILI rate by 1 week for both North and South China. Official ILI rates are typically published with a delay of 1 to 2 weeks, improving further the timeliness of online media data relative to ILI reports.

#### 3.4.9 Miscellaneous surveillance systems

Our search also found several surveillance systems that did not fit into the categories identified in our review.^62^, ^63^, ^64^, ^65^, ^66^ These included systems using body temperature, restaurant reservations, rapid influenza detection tests (RIDT), and an online health support tool.

Bordonaro et al. considered fever data from emergency departments, where fever was defined as body temperature greater than or equal to 38.0°C.^62^ The authors suggested temperature data as a promising surveillance tool as they identified that fever rates provide a stronger signal than some other markers of influenza activity such as flu‐like symptoms. Nsoesie et al. tracked restaurant reservation data from a popular online platform called OpenTable and compared it to ILI data for several cities in the USA and Mexico.^65^ They found a strong correlation between ILI and reservation data for only two cities, with optimal correlations when either no lag or a 1‐week lag was used, suggesting that restaurant reservation data may in some contexts pre‐empt ILI data. While this system required minimal resources and easy access to data, there was too much variability in the results depending on the time and the location that the system was assessing. Temte et al. suggested that their system of testing primary care patients with RIDT followed by immediate, wireless transmission of results to public health authorities correlated strongly with a PCR detection‐based surveillance system.^66^ While the system was able to provide near real‐time surveillance data, a number of limitations hindered the validity of the system.

Two studies looked at usage of a digital decision support and surveillance tool (Thermia).^63^, ^64^ Hswen et al. (2017) found that the peak in ILI reports to Thermia led by approximately 1 month the peak in influenza cases reported by the National Health and Family Planning Commission (NHFPC) of the People's Republic of China. Hswen et al. (2020) primarily investigated the correlation between Thermia usage and COVID cases and also looked at the correlation with percentage positivity data for influenza from the US CDC. In this study, the number of Thermia sessions was found to be negatively correlated with influenza activity, likely because COVID‐19 activity was driving Thermia use.

## 4 DISCUSSION

This scoping review was able to identify and map eight major categories of non‐traditional influenza surveillance systems (and one miscellaneous) with varying levels of evidence. The literature gathered in the review identified that there appears to be no clear agreement on the validity of the various systems that are currently available. While the data appeared to be positive overall, there were disagreements between studies for every data source category. EHR and participatory survey‐based surveillance systems appeared to have the most consistency between the studies. Most of the non‐traditional surveillance systems used data generated for purposes other than respiratory virus surveillance and showed that existing data could be successfully adapted to this new objective without additional data collection. This was greatly facilitated by the increasing availability of electronic data within and outside of the healthcare sector. A common trend identified was that none of the studies suggested a non‐traditional surveillance system as a replacement for the current system. Rather, the non‐traditional systems were proposed as a complement to the current system. In this paper, we focused on publications reporting on single novel sources of data to monitor influenza activity and did not include articles reporting on the use of advanced analytics and modeling approaches to combine sources of data to nowcast and forecast influenza activity. However, we do recognize the potential value of these approaches in improving the use of data from non‐traditional surveillance sources. An example is the work of Davidson et al. who built a network using US CDC sentinel data on laboratory‐confirmed influenza and GFT which produces more reliable real‐time estimates and predictions of influenza cases than using US CDC data alone.^67^

A number of interesting trends were observed in the literature. We found that some of the surveillance systems were more heavily studied in certain geographical locations. For instance, six out of the seven Twitter‐based surveillance system studies were conducted in the USA, where Twitter usage is the most prevalent. Meanwhile, internet search query‐based system studies were found to be more prevalent in China. While we anticipated other types of surveillance systems to be studied more intensely following the invalidation of GFT, our analysis of surveillance systems by data source category and publication year did not show any notable trends (Figure 3).

Practical limitations from an implementation perspective are present for the non‐traditional surveillance systems identified in this review. One of the reasons for the need for a secondary surveillance system is that the current sentinel ILI and SARI systems are not feasible or representative in some geographical locations, leading to gaps in the comprehensiveness of the surveillance coverage^68^, ^69^, ^70^, ^71^, ^72^, ^73^ and low spatial resolution. While many of the non‐traditional systems being developed are certainly lower in cost or build upon existing infrastructures, many require technology and highly trained personnel to successfully implement the systems. For instance, Twitter‐based surveillance systems are reliant on a massive sample size of tweets in a particular region. However, Twitter usage varies significantly by geographical location, and many of the lower resource countries have less access to the internet in general. Therefore, the question remains on whether such complementary system can provide comprehensive and minimally biased surveillance data.

A number of limitations exist with this scoping review. First, as with literature reviews of any type, our search strategy may have missed some relevant studies. This review was limited to studies published after 2007 and only in English, French, or Spanish. However, the decision to consider only studies published after 2007 is unlikely to have had a major impact as the non‐traditional surveillance methods are relatively new and most were studied more intensely following the 2009 influenza pandemic. Likewise, it is important to note that, unlike a systematic review, scoping reviews typically have a broader focus making it difficult to conduct the review without setting a number of reasonable limitations. Second, because this review did not involve critical appraisal of the quality of the evidence of each article, with the exception that they needed to be peer‐reviewed, it is difficult to make practice recommendations or confidently identify gaps in the quality of the literature. This review also did not assess for risk of bias of the literature nor other methodological limitations.

This scoping review mapped and classified the current literature on non‐traditional influenza surveillance systems and found several categories which could serve as complements to traditional surveillance for influenza. In particular, EHR and participatory survey‐based surveillance systems appeared to have the most consistency between the studies identified on their potential for complementing and could merit a more systematic review. Both systems were also used during the COVID‐19 pandemic, and lessons learned from that experience, which were not yet published while this review was done, will be useful for further strengthening surveillance for acute respiratory diseases.

## CONFLICT OF INTEREST

JK is currently an employee of Merck Canada Inc where they receive a salary and own stocks in the following pharmaceutical companies: Pfizer Inc and Eli Lilly. All authors confirm that they did not have any conflicts of interest at the time of their contribution to this work.

## ETHICS STATEMENT

N/A.

## PATIENT CONSENT STATEMENT

N/A.

## PERMISSIONS TO REPRODUCE MATERIAL FROM OTHER SOURCES

N/A.

## AUTHOR CONTRIBUTIONS

Study concept and design: JK, AH, and KV. Acquisition of data: JK, AH, and HS. Analysis and interpretation of data: JK, AH, HS, and KV. Initial drafting of the manuscript: JK. Revision of the manuscript: HS, AH, and KV. Study supervision: AH and KV.

## Supporting information


**Table S1.** Search strategy
**Table S2.** Summary of literature
**Table S3.** Articles by years included in period of study
Each row shows the years included in the period of study for one article. Articles are ordered by year of publication.

## Data Availability

For a full list of included literature, see Table S2 in the supporting information.

## ENDNOTE

*
https://www.covidence.org/home
