# Supplementary material for: Influenza surveillance systems using traditional and alternative sources of data: A scoping review
Source: Influenza Other Respir Viruses. 2022 Sep 8;16(6):965–74. doi: 10.1111/irv.13037 (PMC9530542; doi:10.1111/irv.13037)
Supplement: Supplementary file 1 — Table S1. Search strategy Table S2. Summary of literature Table S3. Articles by years included in period of study Each row shows the years included in the period of study for one article. Articles are ordered by year of publication. [file IRV-16-965-s001.docx]

**Influenza Surveillance Systems Using Traditional and Alternative Sources of Data: A Scoping Review**

**SUPPORTING INFORMATION**

**S1. Search strategy**

| **Database** | **Search Date** | **No. of Articles** | **Search Strategy** |
| --- | --- | --- | --- |
| PubMed | 2 July 2019  28 January 2022 | 139  +44  = 183 | (valid* OR correlat* OR compar* OR concur* OR agree*) AND (influenza OR "influenza-like illness" OR "severe acute respiratory infection") AND (surveillance OR monitor* OR participatory OR crowdsourc* OR "electronic medical record" OR "electronic health record") AND (traditional OR "non-traditional") |
| Web of Science | 2 July 2019  28 January 2022 | 153  +52  = 105 | (valid* OR correlat* OR compar* OR concur* OR agree*) AND (influenza OR "influenza-like illness" OR "severe acute respiratory infection") AND (surveillance OR monitor* OR participatory OR crowdsourc* OR "electronic medical record" OR "electronic health record") AND (traditional OR "non-traditional") |
| Scopus | 2 July 2019  28 January 2022 | 275  +260  = 535 | ( valid* OR correlat* OR compar* OR concur* OR agree* ) AND ( influenza OR "influenza-like illness" OR "severe acute respiratory infection" ) AND ( surveillance OR monitor* ) AND ( participatory OR crowdsource* OR "electronic medical record" OR "electronic health record" ) AND ( traditional OR "non-traditional" ) AND (LIMIT-TO ( PUBYEAR , 2022 ) OR LIMIT-TO ( PUBYEAR , 2021 ) OR LIMIT-TO ( PUBYEAR , 2020 ) OR LIMIT-TO ( PUBYEAR , 2019 ) OR LIMIT-TO ( PUBYEAR , 2018 ) OR LIMIT-TO ( PUBYEAR , 2017 ) OR LIMIT-TO ( PUBYEAR , 2016 ) OR LIMIT-TO ( PUBYEAR , 2015 ) OR LIMIT-TO ( PUBYEAR , 2014 ) OR LIMIT-TO ( PUBYEAR , 2013 ) OR LIMIT-TO ( PUBYEAR , 2012 ) OR LIMIT-TO ( PUBYEAR , 2011 ) OR LIMIT-TO ( PUBYEAR , 2010 ) OR LIMIT-TO ( PUBYEAR , 2009 ) OR LIMIT-TO ( PUBYEAR , 2008 ) OR LIMIT-TO ( PUBYEAR , 2007 ) ) AND ( LIMIT-TO ( DOCTYPE , "ar" ) OR LIMIT-TO ( DOCTYPE , "re" ) OR LIMIT-TO ( DOCTYPE , "cp" ) OR LIMIT-TO ( DOCTYPE , "ed" ) OR LIMIT-TO ( DOCTYPE , "no" ) OR LIMIT-TO ( DOCTYPE , "Undefined" ) ) |

**S2. Summary of literature**

| **Twitter-Based Surveillance Systems** *(n=7)* | | | |
| --- | --- | --- | --- |
| First Author | Geographical location | Years under study | Non-Traditional System Type |
| Allen *et al.* | USA | 2013-2014 | Geographic information system and machine learning methods using Twitter as the data source |
| Aslam *et al.* | USA | 2013-2014 | Twitter posts containing the keyword “flu” in 11 US cities |
| Gesualdo *et al.* | USA | 2012-2013 | Minimally supervised algorithm that learns technical term-naïve pairs from Twitter |
| Nagel *et al.* | USA | 2012-2013 | Twitter posts containing the keywords flu, influenza, pertussis, or whooping cough |
| Velardi *et al.* | USA | 2012-2013 | Twitter mining – authors developed an algorithm to automatically learn a variety of expressions that people use to describe their health conditions. Twitter monitoring instrument was implemented to finely analyze the presence and combinations of symptoms in tweets |
| Volkova *et al.* | USA | 2009-2014 | Expression (affect) signals originating from Twitter surrounding military bases |
| Woo *et al.* | Republic of Korea | 2010-2014 | Twitter and online blog posts (Naver, a popular Korean search engine) |
| **Electronic Health Record-Based Surveillance Systems** *(n=15)* | | | |
| First Author | Geographical location | Years under study | Non-Traditional System Type |
| Aquilar Martin *et al.* | Spain | 2010-2019 | Clinically diagnosed influenza cases from primary care physicians who use the eCAP medical records program |
| Baker *et al.* | USA | 2009 | Combined syndromic and EHR-derived ILI surveillance at UVAHS. Surveillance system included 7 UVAHS sentinel sites and 1 university-based sentinel site. |
| Baltrusaitis *et al.* | USA | 2012-2016 | Patient visits for ILI from cloud-based EHR-based system. |
| Bellazzini *et al.* | USA | 2008-2009 | EHR – chief complaints and ICD-9 discharge diagnoses. |
| Bouzille *et al.* | France | 2010-2015 | eHOP clinical data warehouse – patient data coming from EHRs. |
| Chan *et al.* | Canada | 1998-2003 | Fee-for-service medical billing service. |
| De Oliviera Bernard *et al.* | Australia | 2015-2016 | Electronic medical records from 550 general practices across Australia (MedicineInsight). |
| Hripcsak *et al.* | USA | 2004-2005 | Two systems were developed to identify ILI in ambulatory EHR data from a network of community health centers. The first system used queries on structured data and was designed for this specific EHR. The second used natural language processing of narrative data, but its queries were developed independently from this health records. |
| Keck *et al.* | USA | 2009 | IIAS – an EHR-based surveillance system. |
| Rosenkotter *et al.* | Austria, Belgium, Spain | 2009 | Emergency data-based syndromic surveillance. |
| Schanzer *et al.* | Canada | 2003-2011 | Virological data from FluWatch (PHAC). |
| Thompson *et al.* | Canada | 2008-2011 | EDIS database – real-time ED monitoring system implemented across Winnipeg hospitals that records patient demographics, chief complaints, and other triage info for every ED visit. |
| Viboud *et al.* | USA | 2003-2010 | Electronic medical claims data compiled by IMS Health in 480 USA locations. |
| Yang *et al.* | Taiwan, China | 2014-2017 | Taipei Medical University Health Care System EHRs. |
| Yih *et al.* | USA | 2007-2011 | Weekly aggregate ILI reports from the EHR Support for Public Health (SPH) disease-detection and reporting system – uses an automated algorithm to identify ILI visits among a patient population of about 700,000 in Eastern Massachusetts. |
| **Online Searches and Internet Traffic** *(n=10)* | | | |
| First Author | Geographical location | Years Under Study | Non-Traditional System Type |
| Caldwell *et al.* | USA | 2013-2016 | Internet traffic data from 10 influenza-related webpages on the Centers for Disease Control and Prevention (CDC) website |
| Chang *et al.* | Taiwan, China | 2015-2016 | Online search query data from Google Trends |
| De Toni *et al.* | Italy, Germany, Belgium, and the Netherlands | 2007-2019 | Wikipedia page views for influenza-related pages |
| Dong *et al.* | China | 2013-2014 | Baidu search query assessment for ILI surveillance. |
| Guo *et al.* | China | 2011-2015 | Baidu search engine query data-based prediction model. |
| Hulth *et al.* | Sweden | 2005-2009 | GET WELL application – using search queries originating from the search engine on the medical website Vardguiden.se. |
| Liang *et al.* | China | 2011-2015 | Search queries potentially related to influenza based on Baidu Index, a publicly available search engine database. |
| Ma *et al.* | Sweden | 2009-2013 | A web query algorithm that represents an estimation of nation-wide influenza activity for the previous week. |
| Rohart *et al.* | Australia | 2009-2013 | Internet-based surveillance system. |
| Yuan *et al.* | China | 2009-2012 | Internet search query data from Baidu. |
| **Absenteeism-Based Surveillance Systems** *(n=5)* | | | |
| First Author | Geographical location | Years Under Study | Non-Traditional System Type |
| Barrientos *et al.* | Spain | 2009-2010 | School absenteeism data from kindergarten, primary and secondary public schools. |
| Dong *et al.* | China | 2013-2014 | ILI absenteeism data from primary and secondary schools |
| Duchemin *et al.* | France | 2010-2017 | Weekly number of sick leave spells in the workplace from a large private health insurance company |
| Fan *et al.* | China | 2012-2014 | School absenteeism surveillance. |
| Ma *et al.* | Sweden | 2009-2013 | Aggregated weekday data on school absences due to all-cause illnesses from a private servicing around 50 schools. |
| **Participatory Survey-Based Surveillance Systems** *(n=11)* | | | |
| First Author | Geographical location | Years Under Study | Non-Traditional System Type |
| Ackley *et al.* | USA | 2015-2017 | Temperature readings (oral, rectal, aural or underarm) and symptom reporting (body aches, chills, cough, diarrhea, earache, fatigue, headache, nausea, runny nose, shortness of breath, sore throat, stomach ache) through smart thermometer and mobile application |
| Baltrusaitis *et al.* | USA | 2012-2016 | Reports of ILI from FluNearYou |
| Kalimeri *et al.* | Belgium, Denmark, France, Ireland, Italy, Netherlands, Spain, UK | 2011-2017 | Influenzanet – a platform that shares a common and standardized data collection approach throughout the 9 EU countries involved. |
| Kim *et al.* | Republic of Korea | 2016-2018 | Proportion of influenza diagnoses relative to the total number of any diagnoses submitted to the Fever Coach mobile app for parents and caregivers to manage fever in children. |
| Lwin *et al.* | Singapore | 2016-2018 | Mobile-based surveillance system (FluMob) in healthcare workers requesting users to complete a survey listing 18 acute respiratory symptoms on a weekly basis |
| Marmara *et al.* | Malta | 2014-2016 | Self-reported influenza or ILI through telephone survey. |
| Miller *et al.* | USA | 2015-2018 | Temperature readings from smart thermometers synchronized with smartphones |
| Rehn *et al.* | Sweden | 2011-2013 | Internet-based monitoring system (IMS) – Swedish adaptation of Influenzanet. |
| Tilston *et al.* | UK | 2009 | Online weekly symptom and healthcare-seeking survey (UK flusurvey) |
| Van Noort *et al.* | Netherlands, Belgium, Portugal | 2006-2007 | Gripenet – a fully internet-based system that collects data directly from the population. |
| Zachariah *et al.* | USA | 2013-2014 | Community-based surveillance using text-messaging |
| **Medication Sales-Based Surveillance Systems** *(n=3)* | | | |
| First Author | Geographical location | Years Under Study | Non-Traditional System Type |
| Choi *et al.* | Republic of Korean | 2014-2018 | Weekly rate of ILI-related outpatient claims through nationwide prospective drug utilization monitoring |
| Dong *et al.* | China | 2013-2014 | Data from sales of 62 designated other-the-counter medications used to treat colds and acute respiratory tract infections. |
| Socan *et al.* | Slovenia | 2006-2009 | OTC and prescription drug sales used to treat common colds and ARTI. |
| **Telephone Health Line-Based Surveillance Systems** *(n=4)* | | | |
| First Author | Geographical location | Years Under Study | Non-Traditional System Type |
| Arco Gonzalez *et al.* | Spain | 2011-2013 | Emergency calls made to the EMS call center. |
| Ma *et al.* | Sweden | 2009-2013 | Medical hotline – medical telephone hotline staffed by nurses and owned and operated by county councils across Sweden. |
| McGolrick *et al.* | Canada | 2013-2014 | Self-swabbing surveillance system mediated by a nurse-led THHL in Ontario whereby syndromic surveillance concepts are used to recruit and monitor participants with ILI. |
| Van Dijk *et al.* | Canada | 2004-2006 | Telehealth Ontario – a toll-free helpline provided by the Ministry of Health. |
| **Media Reporting** *(n=2)* | | | |
| First Author | Geographical location | Years Under Study | Non-traditional System Type |
| Li *et al.* | China | 2019-2020 | Fraction of influenza-related microblogs and online news articles collected through a commercial online platform. |
| Yan *et al.* | China | 2009 | Daily news items on the pandemic H1N1 from 8 major popular websites in the province using the keyword “A/H1N1” included in the news item title. |
| **Miscellaneous Surveillance Systems** *(n=5)* | | | |
| First Author | Geographical location | Years Under Study | Non-Traditional System Type |
| Bordonaro *et al.* | USA | 2009-2011 | Fever data from emergency departments |
| Hswen *et al.* | China | 2014-2016 | Online educational tool (Thermia) used to engage parents to answer questions regarding symptoms associated with febrile illnesses. |
| Hswen *et al.* | USA | 2019-2020 | Online educational tool (Thermia) used to engage parents to answer questions regarding symptoms associated with febrile illnesses. |
| Nsoesie *et al.* | USA and Mexico | 2012-2013 | Restaurant reservation data from OpenTable. |
| Temte *et al.* | USA | 2013-2014 | RIDT coupled with immediate, wireless transmission of results to public health entities. |

**S3. Articles by years included in period of study**

Each row shows the years included in the period of study for one article. Articles are ordered by year of publication.

| **Years Under Study** | | | | | | | | | | | | | | | | | | | | | | |
| --- | --- | --- | --- | --- | --- | --- | --- | --- | --- | --- | --- | --- | --- | --- | --- | --- | --- | --- | --- | --- | --- | --- |
| **1998** | **1999** | **2000** | **2001** | **2002** | **2003** | **2004** | **2005** | **2006** | **2007** | **2008** | **2009** | **2010** | **2011** | **2012** | **2013** | **2014** | **2015** | **2016** | **2017** | **2018** | **2019** | **2020** |
|  |  |  |  |  |  |  |  | 1 | 1 |  |  |  |  |  |  |  |  |  |  |  |  |  |
|  |  |  |  |  |  | 1 | 1 | 1 |  |  |  |  |  |  |  |  |  |  |  |  |  |  |
|  |  |  |  |  |  | 1 | 1 |  |  |  |  |  |  |  |  |  |  |  |  |  |  |  |
|  |  |  |  |  |  |  |  |  |  |  | 1 | 1 |  |  |  |  |  |  |  |  |  |  |
|  |  |  |  |  |  |  |  |  |  |  | 1 |  |  |  |  |  |  |  |  |  |  |  |
|  |  |  |  |  |  |  |  |  |  | 1 | 1 |  |  |  |  |  |  |  |  |  |  |  |
| 1 | 1 | 1 | 1 | 1 | 1 |  |  |  |  |  |  |  |  |  |  |  |  |  |  |  |  |  |
|  |  |  |  |  |  |  | 1 | 1 | 1 | 1 | 1 |  |  |  |  |  |  |  |  |  |  |  |
|  |  |  |  |  |  |  |  |  |  |  | 1 |  |  |  |  |  |  |  |  |  |  |  |
|  |  |  |  |  |  |  |  | 1 | 1 | 1 | 1 |  |  |  |  |  |  |  |  |  |  |  |
|  |  |  |  |  |  |  |  |  |  |  |  |  |  | 1 | 1 |  |  |  |  |  |  |  |
|  |  |  |  |  |  |  |  |  |  |  |  |  |  | 1 | 1 |  |  |  |  |  |  |  |
|  |  |  |  |  |  |  |  |  |  |  | 1 | 1 | 1 | 1 |  |  |  |  |  |  |  |  |
|  |  |  |  |  |  |  |  |  |  |  | 1 |  |  |  |  |  |  |  |  |  |  |  |
|  |  |  |  |  |  |  |  |  |  |  |  |  |  | 1 | 1 | 1 |  |  |  |  |  |  |
|  |  |  |  |  |  |  |  |  |  |  | 1 |  |  |  |  |  |  |  |  |  |  |  |
|  |  |  |  |  |  |  |  |  |  |  |  |  |  | 1 | 1 |  |  |  |  |  |  |  |
|  |  |  |  |  |  |  |  |  |  |  |  |  | 1 | 1 | 1 |  |  |  |  |  |  |  |
|  |  |  |  |  |  |  |  |  |  | 1 | 1 | 1 | 1 |  |  |  |  |  |  |  |  |  |
|  |  |  |  |  |  |  |  |  |  |  |  |  |  | 1 | 1 |  |  |  |  |  |  |  |
|  |  |  |  |  | 1 | 1 | 1 | 1 | 1 | 1 | 1 | 1 |  |  |  |  |  |  |  |  |  |  |
|  |  |  |  |  |  |  |  |  | 1 | 1 | 1 | 1 | 1 |  |  |  |  |  |  |  |  |  |
|  |  |  |  |  |  |  |  |  |  |  |  |  |  |  | 1 | 1 |  |  |  |  |  |  |
|  |  |  |  |  |  |  |  |  |  |  | 1 | 1 | 1 | 1 | 1 |  |  |  |  |  |  |  |
|  |  |  |  |  | 1 | 1 | 1 | 1 | 1 | 1 | 1 | 1 | 1 |  |  |  |  |  |  |  |  |  |
|  |  |  |  |  |  |  |  |  |  |  |  |  |  |  | 1 | 1 |  |  |  |  |  |  |
|  |  |  |  |  |  |  |  |  |  |  | 1 | 1 | 1 |  |  |  |  |  |  |  |  |  |
|  |  |  |  |  |  |  |  |  |  |  |  |  |  |  | 1 | 1 |  |  |  |  |  |  |
|  |  |  |  |  |  |  |  |  |  |  | 1 | 1 | 1 | 1 | 1 |  |  |  |  |  |  |  |
|  |  |  |  |  |  |  |  |  |  |  | 1 |  |  |  |  |  |  |  |  |  |  |  |
|  |  |  |  |  |  |  |  |  |  |  |  |  |  |  | 1 | 1 |  |  |  |  |  |  |
|  |  |  |  |  |  |  |  |  |  |  |  |  |  |  | 1 | 1 |  |  |  |  |  |  |
|  |  |  |  |  |  |  |  |  |  |  |  |  | 1 | 1 | 1 | 1 | 1 |  |  |  |  |  |
|  |  |  |  |  |  |  |  |  |  |  |  |  |  |  |  | 1 | 1 | 1 |  |  |  |  |
|  |  |  |  |  |  |  |  |  |  |  |  |  |  |  | 1 | 1 |  |  |  |  |  |  |
|  |  |  |  |  |  |  |  |  |  |  | 1 | 1 | 1 | 1 | 1 | 1 |  |  |  |  |  |  |
|  |  |  |  |  |  |  |  |  |  |  |  |  |  | 1 | 1 | 1 | 1 | 1 |  |  |  |  |
|  |  |  |  |  |  |  |  |  |  |  |  | 1 | 1 | 1 | 1 | 1 | 1 |  |  |  |  |  |
|  |  |  |  |  |  |  |  |  |  |  |  |  | 1 | 1 | 1 | 1 | 1 |  |  |  |  |  |
|  |  |  |  |  |  |  |  |  |  |  |  | 1 | 1 | 1 | 1 | 1 |  |  |  |  |  |  |
|  |  |  |  |  |  |  |  |  |  |  |  |  | 1 | 1 | 1 |  |  |  |  |  |  |  |
|  |  |  |  |  |  |  |  |  |  |  |  |  | 1 | 1 | 1 | 1 | 1 | 1 | 1 |  |  |  |
|  |  |  |  |  |  |  |  |  |  |  |  |  |  |  |  | 1 | 1 | 1 | 1 |  |  |  |
|  |  |  |  |  |  |  |  |  |  |  |  |  |  |  |  |  | 1 | 1 | 1 | 1 |  |  |
|  |  |  |  |  |  |  |  |  |  |  |  |  |  |  |  |  |  | 1 | 1 | 1 |  |  |
|  |  |  |  |  |  |  |  |  |  |  |  |  |  |  | 1 | 1 | 1 | 1 |  |  |  |  |
|  |  |  |  |  |  |  |  |  |  |  |  |  |  |  |  |  |  |  |  |  | 1 | 1 |
|  |  |  |  |  |  |  |  |  |  |  |  |  |  |  |  |  | 1 | 1 |  |  |  |  |
|  |  |  |  |  |  |  |  |  |  |  |  |  |  |  |  |  | 1 | 1 | 1 |  |  |  |
|  |  |  |  |  |  |  |  |  |  |  |  |  |  |  |  |  |  | 1 | 1 | 1 |  |  |
|  |  |  |  |  |  |  |  |  |  |  |  |  |  |  |  |  | 1 | 1 | 1 |  |  |  |
|  |  |  |  |  |  |  |  |  |  |  |  | 1 | 1 | 1 | 1 | 1 | 1 | 1 | 1 |  |  |  |
|  |  |  |  |  |  |  |  |  |  |  |  |  |  |  |  | 1 | 1 | 1 |  |  |  |  |
|  |  |  |  |  |  |  |  |  |  |  |  |  |  |  |  | 1 | 1 | 1 | 1 | 1 |  |  |
|  |  |  |  |  |  |  |  |  | 1 | 1 | 1 | 1 | 1 | 1 | 1 | 1 | 1 | 1 | 1 | 1 | 1 |  |
|  |  |  |  |  |  |  |  |  |  |  |  |  |  |  |  |  |  |  |  |  | 1 | 1 |
|  |  |  |  |  |  |  |  |  |  |  |  | 1 | 1 | 1 | 1 | 1 | 1 | 1 | 1 | 1 | 1 |  |
